# Field Evaluation of Promising Indigenous Entomopathogenic Fungal Isolates against Red Palm Weevil, *Rhynchophorus ferrugineus* (Coleoptera: Dryophthoridae)

**DOI:** 10.3390/jof9010068

**Published:** 2023-01-02

**Authors:** Koko D. Sutanto, Ibrahim M. Al-Shahwan, Mureed Husain, Khawaja G. Rasool, Richard W. Mankin, Abdulrahman S. Aldawood

**Affiliations:** 1 Department of Plant Protection, College of Food and Agriculture Sciences, King Saud University, P.O. Box 2460, Riyadh 11451, Saudi Arabia; 2 USDA Agricultural Research Service, Center for Medical, Agricultural, and Veterinary Entomology, Gainesville, FL 32608, USA

**Keywords:** biological control, *Rhynchophorus ferrugineus*, date palm injection, acoustic sensor, sound signal, burst impulses

## Abstract

The rate of the sounds (i.e., substrate vibrations) produced by the movement and feeding activity of red palm weevil (RPW) pest infestations in a date palm tree was monitored over time after trees were separately treated with injection of entomopathogenic fungal isolates, *Beauveria bassiana* and *Metarhizium anisopliae,* or water treatment as the control. The activity sensing device included an accelerometer, an amplifier, a digital recorder, and a signal transmitter that fed the data to a computer that excluded background noise and compared the rates of bursts of movement and feeding sound impulses among treated trees and controls. Observations were made daily for two months. The rates of bursts were representative of the feeding activity of RPW. The unique spectral pattern of sound pulses was typical of the RPW larval feeding activity in the date palm. The microphone confirmed that the same unique tone was produced in each burst. Two months after fungal injection, the RPW sound signal declined, while the RPW sound signal increased in the control date palms (water injection). The mean rates of bursts produced by RPW decreased to zero after the trees were injected with *B. bassiana* or *M. anisopliae* compared with the increased rates over time in the control treatment plants.

## 1. Introduction

The red palm weevil (RPW), *Rhynchophorus ferrugineus* (Olivier, 1790) (Coleoptera: Dryophthoridae), is a serious pest of date palms in Saudi Arabia and was first reported in Al-Qatif Governorate in 1987 [1]. An estimated average annual loss of USD 2–9 million has been reported in Saudi Arabia for the removal of infested palms [2]. The larval stage destroys internal tissues and causes the death of date palms [1]. Various tactics have been used to control RPW, such as pheromone traps [3,4,5,6,7,8,9] and microbial agents such as fungi [10,11,12,13,14,15,16]. The entomopathogenic fungi *Beauveria bassiana* (Bals.-Criv.) Vuill. (1912) (Hypocreales: Cordycipitaceae) and *Metarhizium anisopliae* (Metschn.) Sorokīn. (1883) (Hypocreales: Clavicipitaceae) caused 95% mortality of adult RPW when applied under laboratory conditions via an auto-contamination trap [13]. In another study, *B. bassiana* and *M. anisopliae* caused 80–82% mortality in eggs and hatched larvae, while adult mortality reached up to 100% within 2–3 weeks [10]. M. anisopliae showed substantial mortality of RPW that was greater than 90% within seven days at a concentration of 3 × 10^8^ spores/mL [17]. In addition, *B. bassiana* and *M. anisopliae* were tested against the second and fourth instars of RPW, which resulted in significant mortality as well as decreased egg hatchability, pupation duration, and adult emergence rates [18]. RPW larvae were infected by both *B. bassiana* and *M. anisopliae*; however, only *B. bassiana* led to a high mortality rate of 93.3% after 21 days, whereas *M. anisopliae* only resulted in 66.7% [19].

The application of fungal species to infect the RPW larval stage has been investigated in date palms in greenhouses using acoustic sensors [20]. Acoustic technology is frequently applied to detect substrate-borne vibrations of larvae that move and feed in a tree trunk [21]. Feeding and movement activity detected using the measurements of substrate vibrations produced by hidden RPW larvae can be distinguished from background noise by comparing the frequency spectra and times of occurrence of their sound impulse bursts with similarly obtained measurements in which the presence of larvae has been experimentally confirmed [20,22,23,24,25,26]. The control efficacy of the fungus can be followed by monitoring the reductions in the rates of bursts of impulses produced by RPW larvae after artificial infestation inside the date palm offshoot [20]. The characteristics of the spectral profiles produced by RPW larval activity depend on the sizes of the larvae, the sizes of the trees, and the distance between the acoustic sensor and the larval activity point in the tree [27,28,29]. Related studies that include the consideration of the rates of bursts of larval feeding impulses have also been conducted on palmetto weevil, *Rhynchophorus cruentatus* (Fabricius, 1775) (Coleoptera: Scarabaeidae) [28], and fruit stalk borer, *Oryctes elegans* (Prell, 1914) (Coleoptera: Scarabaeidae) [30]. In this study, the pathogenicity of fungal isolates against RPW was investigated in RPW naturally infested date palm trees with the injection of entomopathogenic fungal isolates, and the efficacy of the isolates in causing reduced rates of RPW sound impulse bursts was monitored with an acoustic sensor.

## 2. Materials and Methods

### 2.1. Study Site

A field experiment was conducted during the period that began in February 2021, in a date palm orchard located in Al–Kharj Governorate, Riyadh Region, Kingdom of Saudi Arabia (24°16′21.87″ N, 47°12′16.63″ E). The date palm variety in this farm was Khalas. The farm had been infested by RPW, according to confirmation from the farm manager. Date palms that were medium in size (ranging 3–4 m in height) and similar in age (around 15–16 years) were selected. Visual symptoms included evidence of tunnels in the trunk of the date palm around the base, which contained chewed plant tissue and an odor of fermentation, as well as the presence of brown liquid material. Furthermore, tree infestation was confirmed using an acoustic sensor to detect the RPW sound signal inside the trees.

### 2.2. Entomopathogenic Fungal Source

Two fungal isolates that were isolated from RPW in Saudi Arabia and were previously identified (Table 1) were injected into date palms as treatment in comparison with a water control in a field experiment.

### 2.3. Mass Production of Fungal Isolates

Fungal isolates successfully grown in potato–dextrose–agar (PDA) medium were selected for mass production using wheat medium [13,31]. The first step was weighing 1 kg of wheat, which was washed in tap water and then rewashed with distilled water. Wheat was kept for one night in the plastic container; then, the wheat was placed on an aluminum foil and autoclaved. Autoclaved wheat was inoculated with a small portion of each fungal isolate (1 cm) and spread out on wheat medium. Inoculated wheat was incubated for 2–3 weeks at 28 ± 1 °C with 80 ± 5% relative humidity under dark conditions, which facilitated optimal growth for fungal sporulation. After germination, the wheat medium was transferred to a sterile plastic box, and the cover boxes were opened and dried for one day in the incubator. Dry fungi were collected and then ground to powder.

### 2.4. Date Palm Injection Procedures

In preparing the fungal solution for date palm injection, the fungal concentration was determined under a microscope with a hemocytometer. In this experiment, the concentration was used similarly to a previous experiment under laboratory conditions, which was 1 × 10^9^ conidia/mL [16]. Five liters of each fungal solution were prepared and kept in a plastic container. Distilled water was used for the control treatment. Then, each fungal solution was placed in a special balloon (Ynject, Fertinyect, S.L, Córdoba, Spain) by filling a special syringe of 20 mL (Henke–Sass, Wolf, Tuttlingen, Germany) and inserting it into the balloon five times for date palm injection.

### 2.5. Preliminary Study of the Fungal Treatment Effects

A preliminary experiment was performed by injecting each fungal solution (BbSA-1 or MaSA-1) into the infested date palm trees in three replicates in Al–Ammariah, near Riyadh, KSA. The effect of the fungal treatment was demonstrated 45 days after injection. The date palms were dissected lengthwise 1 m in length. Each meter log was cut into two equal halves and then into four quarters for more detailed internal observations. Each quarter was observed to record any signs of RPW infestation (destroyed tissue) and to collect dead RPW individuals. RPW larva mortality inside the date palm trunk was 100% after fungal injection in all trees. The dead RPW larvae were collected and transferred into sterile Petri dishes with moist filter paper and incubated at 28 ± 1 °C with 80 ± 5% RH under laboratory conditions. After 2–6 days of incubation of the dead larvae, the first fungal symptom that covered the RPW larval body appeared. Based on this preliminary study, the use of acoustic sensors was adopted as the preferred method for monitoring treatment effects, because palm dissection is a time-consuming, expensive, and destructive procedure for assessing treatment effectiveness in the field [24].

### 2.6. Trunk Injection

**Figure 1:**
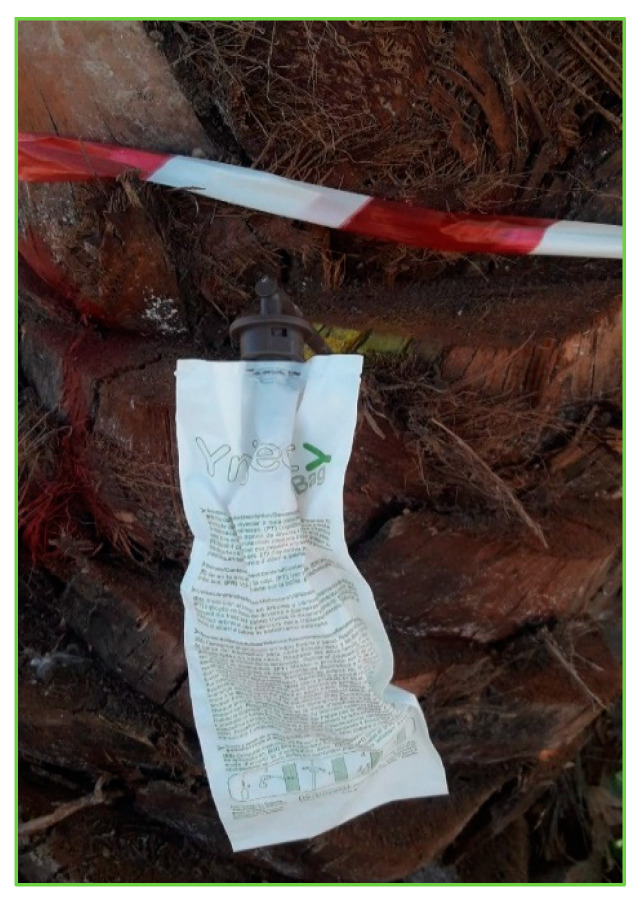
A balloon filled with fungal solution was inserted into each hole in the date palm.

The selected date palms were injected with fungal treatment or water control solution at multiple locations per tree using a balloon injector as follows: Four holes were drilled in a clockwise spiral around each date palm trunk, each pointed downward at a 30° angle, using a drill machine equipped with a brad point drill bit (diameter, 8 mm). The points of injection were 25, 50, 75, and 100 cm above the ground level. Immediately after drilling, the fungal solution was delivered to the trunk. Each balloon injector with 100 mL of the solution was inserted into each hole (Figure 1) for a total injection volume of 400 mL of fungal solution. In the control treatment, date palms were injected with sterile distilled water. All treatments were injected on the same day.

### 2.7. Temperature of Infested Date Palms

Temperatures were measured inside and outside each treated tree during the two months of acoustic data collection using Elitech Digital Thermometer Hygrometer Dt-2 (Maoming, China).

### 2.8. Acoustic Sensor “TreeVibes”

**Figure 2:**
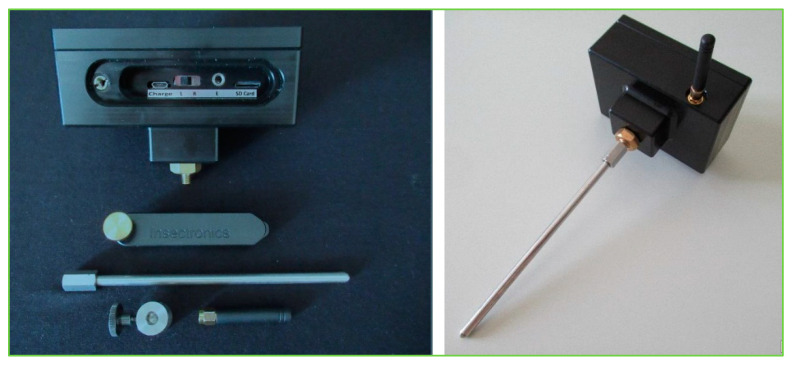
The main black box on the **left** is the recorder, which contained the secure digital (SD) card, antenna for communication, adapter, and waveguide; the acoustic sensor assemble is shown on the **right** (TreeVibes manual, Insectronics.net (accessed on 15 January 2020)).

The RPW larval activity vibrations were monitored with a TreeVibes acoustic detection device (Insectronics, Insect Surveillance Technology, Crete, Greece) that included an accelerometer sensor; associated probes and adapters; and a protective box with an amplifier, headphone output, and a digital recorder. The latter contained a secure digital (SD) card, power supply, rechargeable battery and solar panel, and a signal transmitter system with an antenna (Figure 2). The amplified signal was fed to both the recorder and the headphone output, enabling the user to assess the signal during the recording process. This and similar devices are routinely used for the detection of vibrations produced by wood-boring insects inside their host over distances of up to 2–4 m [32].

### 2.9. Acoustic Sensor Placement and Vibrational Signal Collection Procedures

**Figure 3:**
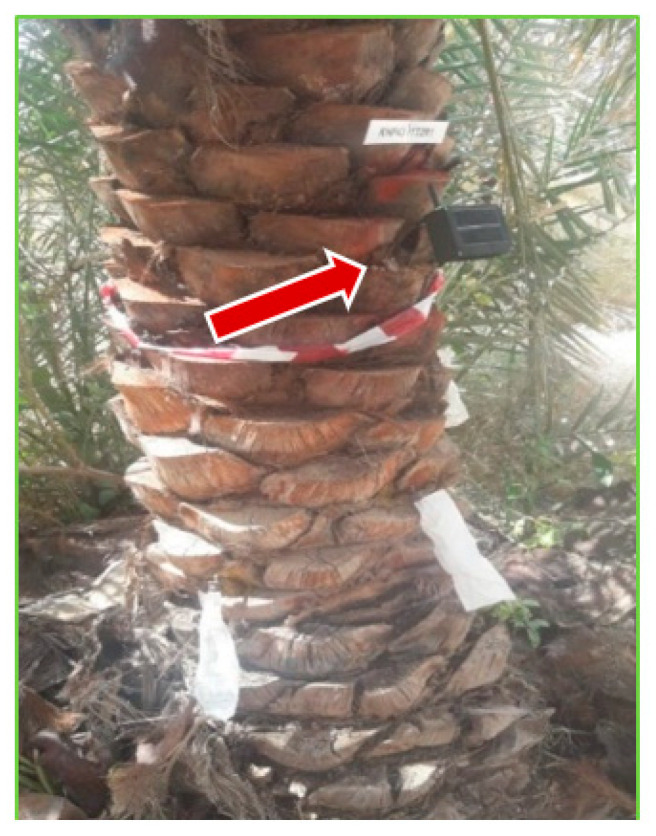
The installed acoustic sensor on a date palm trunk. The arrow is showing the device probe inserted inside the date palm tree trunk.

Preliminary recordings of insect activity were collected from each date palm before its injection with treatment or control solution. The reading points were marked on each date palm trunk (one meter above the ground). A hole was drilled into the trunk using the brad point drill bit previously used to insert holes for fungal injection; a 35 cm waveguide probe was inserted into the hole, and the TreeVibes device was attached to the probe (Figure 3). For the preliminary and the treatment comparison experiments, a timing protocol was set up to record four periods of 20 s daily at each date palm recording site over 24 h (00.00–7:30, 7:30–12:00, 12:00–19:30, and 19:30–24:00); the recordings were timestamped and then forwarded to a remote website. Periodically, the sound data were manually downloaded from the website and then stored permanently on a PC (Dell; OPTIPLEX 7010) in the laboratory.

### 2.10. Signal Processing

Raven Pro interactive sound analysis software version 1.5 [33] was used to observe and screen the sound recordings, which also were analyzed using DAVIS insect sound analysis program [27], which compared the spectral characteristics and timing of each train of impulses in the 20 s samples with the mean characteristics of impulse bursts previously verified to be produced by that species (Mankin, 2011). The spectral characteristics of the insect sounds in the preliminary recordings were confirmed to be different from the background noise both in Raven and DAVIS. In addition, the last instar of RPW larvae produces a unique squealing sound [29], which frequently occurred in these recordings. Background noises such as bird calls, wind, and vehicular operations could be readily differentiated from insect sound bursts due to differences in their frequency spectra and impulse timing patterns [21].

### 2.11. Statistical Analysis

The experiment had a randomized complete block design with 12 replicates for each treatment. Analyses of variance were conducted using SAS (SAS 2004) for the comparisons of mean differences between temperatures measured outside and inside each date palm and mean rates of impulse bursts for different treatments. Means were separated using the Least Significant Difference (LSD) test at *p* < 0.05.

## 3. Results

### 3.1. Ambient and Inside Temperatures of an Infested Date Palm

Temperatures were measured in the treated and controlled date palms. The temperature values inside the date palm treated with *B. bassiana* and *M. anisopliae* were not significantly different (Table 2). The temperatures inside the date palm were significantly different between treated and control trees.

### 3.2. Sound Recording of RPW Activity in a Date Palm

**Figure 4:**
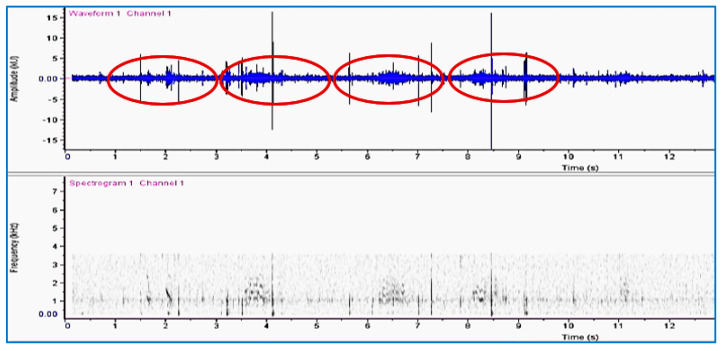
Oscillogram (waveform) and spectrogram patterns produced by activity of red palm weevil larvae inside a water-injected date palm. Three series of high-amplitude feeding activity clicks mixed with bursts of low-amplitude movement impulses are marked with ovals.

An oscillogram and spectrogram of signals recorded in the infested date palms before treatment with fungal isolates is shown in Figure 4. The rate of impulse bursts was characteristic of the typical feeding activity of RPW larvae.

**Figure 5:**
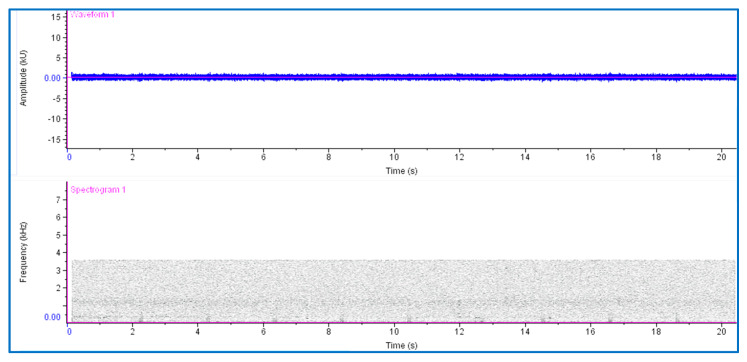
The oscillogram and spectrogram revealed that no sound signals were produced by RPW larval activity two months after fungal injection, unlike the signal patterns from control treatment palms.

**Figure 6:**
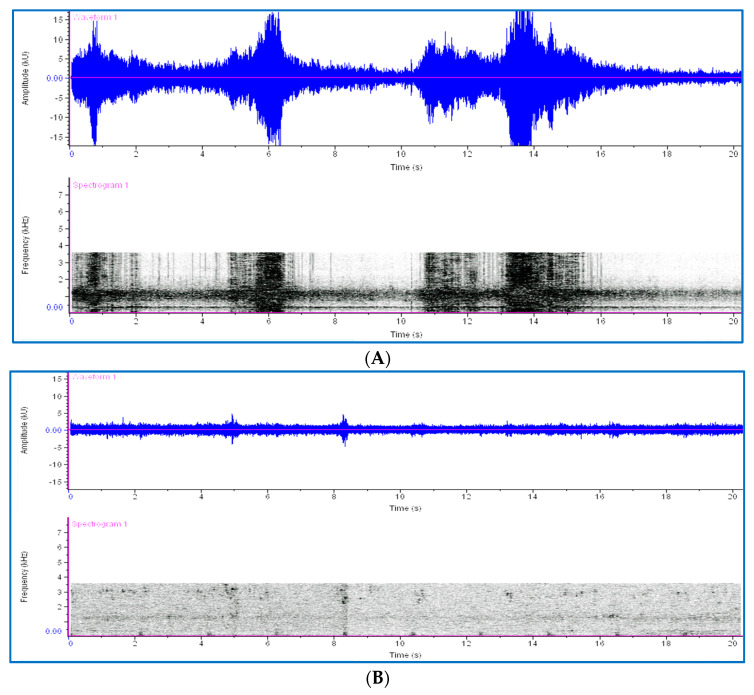
Comparison of background noise spectrograms in the natural environment: (**A**) spectrograms for wind noise and (**B**) spectrograms for bird noise in the environment.

The sounds generated by RPW larval feeding activity in date palms were detected as distinct spectral patterns during recording, with the generated sounds being considered as background sounds. The spectral profiles of RPW larval sounds were flattened when the date palm was sprayed with fungi (Figure 5); in addition, other background sounds such as wind and bird calls could be clearly distinguished (Figure 6). No other insect borer was found on the farm during this study.

**Figure 7:**
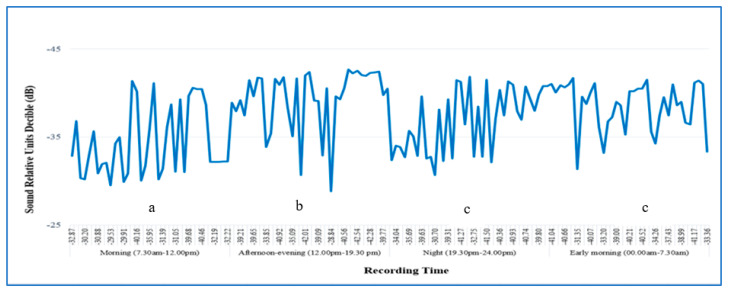
The relative spectral amplitudes (dB) of the red palm weevil larval sound signal were recorded inside the infested date palms for 24 h—early morning (00:00–7:30), morning (7:30–12:00), afternoon (12:00–19:30), and night (19:30–24:00).

**Figure 8:**
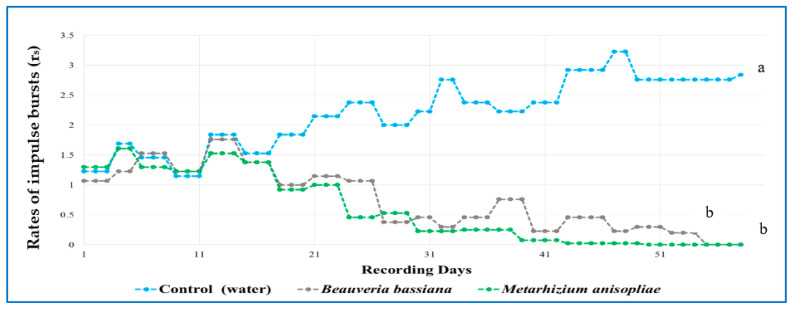
The mean rates of bursts per day of sound recording produced by red palm weevil larval activity inside the infested date palms is shown from the first day of injection to the last day of recording in treated trees and in control trees.

The relative amplitudes (dB) of the RPW larval sounds were recorded for 24 h in an infested date palm, and the signals were divided into morning, afternoon, night, and early-morning periods (Figure 7). The purpose was to detect red palm weevil actively feeding. A significant difference was found in the relative amplitudes (dB) of signals produced by RPW larvae at different times of recording within 24 h. Two months after fungal injection, the sound of RPW larval activity in the infested date palm declined, while the sound of RPW larval activity in the control tree increased. The average number of burst pulses generated by RPW larval activity was zero in the date palms injected with *B. bassiana* or *M. anisopliae* (Figure 8). By comparing the mean burst rates daily for each treatment, a significant difference was observed in the mean rates of impulse bursts in the treated trees compared with the control trees by the end of the experiment.

## 4. Discussion

The application of microbial agents, especially entomopathogenic fungi, can be a new strategy to control insect borers [34]. The present study demonstrated that the application of both entomopathogenic fungal isolates showed zero RPW activity after injection into naturally infested date palm trees. Our results are supported by other research studies where the application of *B. bassiana* through trunk injection suppressed RPW by up to 90% in the field [35,36]. Conidial suspensions of *B. bassiana* and *M. anisopliae* showed high survival rates when injected into coconut seedlings against *Rynchophorus palmarum* (Linnaeus, 1758) (Coleoptera: Dryophthoridae) [37]. The efficacy of applying microbial agents to palm trees to control insect borers can be easily assessed using acoustic sensors to detect the presence of insect borers in the trees [38]. The recording of RPW activity sounds inside date palms can be used to evaluate the fungal treatment of infested date palms. Here, RPW larval activity (sound signals) inside date palms differed significantly between fungal and control treatment palms. Other studies confirmed the sound signal produced by RPW larvae inside palms [21,25,27,29,39]. An acoustic sensor produced sound recordings of RPW larvae within infested coconut palms, with the lowest frequency band being 500–1000 Hz, while no significant sounds were detected in noninfested trees [40]. In this study, the number of burst pulses in the spectral profile generated by larval activity was similar to the pattern of the spectral profile in another study [20]. A series of pulses (vertical “clicks”) between and within bursts is most likely caused by larvae snapping and scraping the surface inside the palms [21].

The use of an acoustic sensor to detect RPW activity in treated and control date palms showed that larval activity in the treated date palms was greatly reduced after two months compared with the water-injected date palm trees, indicating that the fungi greatly reduced the feeding and movement activity of RPW larvae. This result was confirmed by another study that found that RPW activity in *B. bassiana*-treated trees was significantly lower than that in control trees [24]. It is possible that an infected RPW larva inside the date palm was the cause; infected larvae typically move slowly and are weaker [41,42]. In this study, naturally infested date palms were used, so we did not know the age of RPW larvae in the palm trunks, and the acoustic sensor we used was limited in determining the exact larval stage in the date palm. It has been mentioned that the age of RPW larvae in field studies cannot be determined with acoustic measurements [24].

In this study, no other insect activity interfered with the recording of the target insect (RPW), nor were there other insect signs around the date palm recording site. Moreover, the spectral profile pattern, which is most likely the sound signal generated by RPW larval activity inside the date palm, could be distinguished from many other background sounds. In contrast, a study conducted in Malaysia on oil palms using an acoustic sensor to identify RPW activity showed the presence of RPW and termites (Blattodea: Termitidae) in the trees. However, the spectral profiles generated by termite and RPW activity were different [25]. Infested trees have a different spectral profile and frequency variation because the different insect pests have different characteristics in many pulse sequences, while noninfested stems have a flattened spectral profile [32]. In addition, RPW activity inside palms can be detected using an acoustic sensor. According to a previous study, the early and late stages of RPW have similar spectral profiles, and early-stage larvae can be detected at a distance of 0.5–1 m from the infestation source, while late-stage larvae can be detected at a distance of 1–4 m [21]. In addition, RPW larvae of all instars can be detected at a distance of 5–10 cm under controlled or open field conditions [29].

The acoustic sensor used in this study was sensitive enough to detect the sound of feeding and the movement of RPW larvae within date palms. This was confirmed by the results of another study in which other acoustic sensors were used. Another acoustic sensor, IoTree Smart, was evaluated to detect the sound signal of RPW larvae in coconuts and oil palms in Malaysia [25]. Similarly, a Postharvest insect Detection System (PDS) with an electret sensor was tested to detect another insect pest, rice weevil, *Sitophilus oryzae* (Linnaeus, 1763) (Coleoptera: Curculionidae) in stored products [43]. Acoustic sensors, and insect movement and feeding behavior vibration analyses could improve future early detection and biological control applications [38].

This study was conducted during the period from winter to spring in 2021, and the minimum and maximum temperatures inside and outside the date palm trees varied (inside, 18–28 °C; outside, 15–40 °C), which might have supported fungal activity inside the palm trees, and it is believed that the fungus still caused infection in the host organism. In another study, a fungal isolate was found to have high infectivity against wax moth, *Galleria mellonella* (Linnaeus, 1758) (Lepidoptera: Pyralidae), between 15 and 30 °C, but low infectivity at 10 °C [44]. *B. bassiana*, *M. anisopliae*, or *Paecilomyces fumosoroseus* (Wize) A.H.S. Br. & G. Sm. (1957) (Hypocreales: Cordycipitaceae) did not infect *G. mellonella* at 35 °C; however, infection occurred when fungi were present at the low temperatures of 10–20 °C [45]. It was reported that a fungal isolate still had a germination rate of 31.3% at a low temperature (8 °C). However, 29–92% germination was achieved in PDA medium in over 10 days under laboratory conditions [46].

The obtained results also clearly show the effectiveness of injecting fungal isolates into a date palm under field conditions, and an acoustic sensor can be used to demonstrate the efficacy of fungal isolates. The presence of the RPW larval gallery created by feeding the RPW larvae may have allowed the fungus to penetrate the palm tissue after injection, and the use of injection methods to introduce the fungus into infested date palms for an IPM program in the field is promising.

## 5. Conclusions

The efficacy of the fungal isolates used in this field study using the injection system against RPW is promising. The acoustic sensor could be used to monitor the RPW sound signal within the date palm trunks during fungal treatment. In this study, the local fungal isolate of *Metarhizium anisopliae* proved to be better than the *Beauveria bassiana* isolate. The larval activity was measured using the acoustic sensor in terms of the rate of impulse bursts; it declined faster for *M. anisopliae* than for *B. bassiana*. These fungal isolates can persist inside the date palm trees for several months and have the potential to protect date palm trees from RPW attack.

## Figures and Tables

**Table 1:** Names and origins of entomopathogenic fungal isolates used in this study.

Isolate Name	Isolate Code	Insect Species/ Order	Source of Isolation	Origin	Coordinates
*Metarhizium anisopliae*	MaSA–1	Red palm weevil/Coleoptera	Adult	Riyadh, Saudi Arabia	N: 24.41867°; E: 46.65408°
*Beauveria bassiana*	BbSA–1	Red palm weevil/Coleoptera	Adult	Al Qatif governorate, Saudi Arabia	N: 26.34437°; E: 43.69217°

**Table 2:** Temperatures in treated and control trees.

Treatment *	Ambient Temperature (°C) **	Temperature inside Date Palm (°C) **
Control (water)	19.93 ± 0a	27.07 ± 0a
*B. bassiana* (BbSA–1)	19.70 ± 0.23a	23.66 ± 0.26b
*M. anisopliae* (MaSA–1)	19.93 ± 0a	24.19 ± 0.23b

* Treatment of fungal isolates injected with *Beauveria bassiana* (BbSA-1) or *Metarhizium anisopliae* (MaSA-1) Saudi Arabian isolates (SA), or distilled water as a control. ** Means followed by the same letter in the same column were not significantly different at α: 0.05.

## Data Availability

All relevant data are within the paper.
